# Amlexanox inhibits production of type I interferon and suppresses B cell differentiation in vitro: a possible therapeutic option for systemic lupus erythematosus and other systemic inflammatory diseases

**DOI:** 10.1136/rmdopen-2024-005351

**Published:** 2025-05-07

**Authors:** Albin Björk, Mohamed Javad Wahadat, Maria Sánchez-Blázquez, Merijn Braams, Jasper Rip, Sander J van Tilburg, Cornelia G van Helden-Meeuwsen, Sylvia Kamphuis, Thierry P P van den Bosch, Zana Brkic, Marjan A Versnel

**Affiliations:** 1 Department of Immunology, Erasmus MC University Medical Center Rotterdam, Rotterdam, The Netherlands; 2 Department of Medicine Solna, Karolinska Institutet, Stockholm, Sweden; 3 Center for Rheumatology, Academic Specialist Center, Stockholm, Sweden; 4 Department of Pediatric Rheumatology, Sophia Children’s Hospital, Erasmus MC University Medical Center Rotterdam, Rotterdam, The Netherlands; 5 MS Center ErasMS, Erasmus MC University Medical Center Rotterdam, Rotterdam, The Netherlands; 6 Department of Pathology and Clinical Bioinformatics, Erasmus MC University Medical Center Rotterdam, Rotterdam, The Netherlands; 7 Department of Internal Medicine, Division of Clinical Immunology and Allergology, Erasmus MC University Medical Center, Rotterdam, The Netherlands

**Keywords:** Sjogren's Syndrome, Lupus Erythematosus, Systemic, Scleroderma, Systemic, Treatment

## Abstract

**Objectives:**

Activation of the type I interferon (IFN) pathway and autoreactive B cells are key immunopathogenic features of systemic lupus erythematosus (SLE), primary Sjögren’s disease (pSjD) and systemic sclerosis (SSc). TANK-binding kinase 1 (TBK1) is a mediator of type I IFN and essential during B cell development in mice. We investigated the properties of the TBK1 inhibitor amlexanox in systemic autoimmune diseases.

**Methods:**

The effects of amlexanox on peripheral blood mononuclear cells (PBMCs) stimulated with Imiquimod, CpG-A, Poly:IC, G3-YsD and 3p-hpRNA were assessed. B cells from healthy controls and patients with SLE, pSjD and SSc were cultured with CD40L, IL-21, IFN, B cell activating factor (BAFF) and amlexanox. Differentiation into CD38^high^CD27^high^CD138^+/-^ cells, proliferation, and IgM and IgG production were measured.

**Results:**

Amlexanox inhibited production of type I IFN induced through endosomal and cytosolic routes in PBMCs. Likewise, supernatants from amlexanox-treated cells did not induce expression of *BAFF* and *MX1*. Amlexanox inhibited spontaneous *MX1* expression in PBMCs from SLE, pSjD and SSc patients. Immunohistochemical staining confirmed expression of the TBK1 protein in pSjD salivary glands. Using a B cell differentiation assay, addition of amlexanox decreased B cell proliferation and differentiation into CD27^high^CD38^high^CD138^+/-^ plasmablasts and plasma cells. Correspondingly, production of IgM and IgG was suppressed. The observations were corroborated in B cells from patients with SLE, pSjD and SSc.

**Conclusions:**

Our findings demonstrate inhibitory effects of amlexanox on type I IFN production and B cell differentiation in primary human cells. Inhibition of TBK1 could potentially be a therapeutic option for the treatment of type I IFN-driven systemic inflammatory diseases.

**WHAT IS ALREADY KNOWN ON THIS TOPIC**

- Amlexanox is a small molecule TANK-binding kinase 1 inhibitor with good tolerability in humans.
- TANK-binding kinase 1 mediates type I interferon production and is implicated in B cell functions.

**WHAT THIS STUDY ADDS**

- We found inhibitory effects of amlexanox on type I interferon production as well as on differentiation and proliferation of B cells *in vitro*.

**HOW THIS STUDY MIGHT AFFECT RESEARCH, PRACTICE OR POLICY**

- Amlexanox might be a promising novel drug for systemic rheumatic diseases with a high type I interferon expression.

## Introduction

 There is a lack of effective drugs in the therapeutic arsenal for the systemic autoimmune diseases systemic lupus erythematosus (SLE), primary Sjögren’s disease (pSjD) and systemic sclerosis (SSc). In these diseases, most patients share the immunophenotypic characteristic of systemic type I interferon (IFN) pathway activation.^1^ Likewise, patients generally exhibit hyperactivity of B cells resulting in autoantibody production and increased risk of B cell lymphomas.^2 3^ Production of type I IFNs can be induced by endosomal Toll-like receptors (TLRs) as well as by cytosolic RNA-sensing RIG-I-like receptors (RLRs) and DNA-sensing receptors (DSRs). Moreover, type I IFNs stimulate differentiation and survival of B cells directly and indirectly through IFN-induced production of B cell activating factor (BAFF). Although the different clinical phenotypes, the immunophenotypic similarities between SLE, pSjD and SSc suggest the applicability of similar treatment approaches. In SLE, the monoclonal antibodies belimumab (targeting BAFF) and anifrolumab (targeting the IFN-α/β receptor (IFNAR)) are already being used but cannot relieve all patients from symptoms and complications.^4 5^ Drugs that target molecules upstream of type I IFN production, and thereby also BAFF production, could be more effective.

Tumour necrosis factor (TNF) receptor-associated factor NF-κB activator (TANK)-binding kinase 1 (TBK1) is a serine/threonine kinase primarily known for its role in innate immunity and antiviral responses. As a member of the non-canonical IκB kinase (IKK) family, TBK1 shares sequence homology with the closely related kinase IKKε. Both molecules require phosphorylation at serine 172 to become activated. TBK1 mediates phosphorylation of interferon regulatory factor (IRF)3 and IRF7 downstream of several pattern recognition receptors (PRRs), ultimately leading to production of type I IFN. As such, TBK1 is a key signalling component in several pathways implicated in systemic autoimmunity, and thus a potential therapeutic target.

Beyond the functions of TBK1 in innate immunity, emerging data highlight its importance also in adaptive immunity. B cell intrinsic TBK1 was recently found to be essential for germinal centre (GC) formation in mice by regulating the balance of the transcription factors IRF4 and B cell lymphoma 6 (BCL6).^6^ Furthermore, TBK1 interacts with the inducible T-cell costimulator (ICOS) to promote the development of T follicular helper cells.^7^ A role for TBK1 in isotype switching to IgA has also been described.^8^

Amlexanox is a selective inhibitor of TBK1/IKKε used mostly in Japan for the treatment of asthma, allergic rhinitis and aphthous ulcers. It is a small molecule with high stability, a shelf life of 3 years and low toxicity, which can be well tolerated by patients when taken as oral tablets.^9^ Interestingly, amlexanox has shown beneficial effects on metabolic dysfunction in mice,^10 11^ as well as improved glucose control in patients with type 2 diabetes.^12^ We hypothesise that amlexanox can be useful for the treatment of type I IFN positive systemic autoimmune diseases.

Here, we analyse whether amlexanox can lower type I IFN production and IFN-induced *BAFF* expression in primary human peripheral blood mononuclear cells (PBMCs) stimulated with ligands for TLRs, RLRs and DSRs. We assess TBK1 expression in patient tissue and evaluate the effect of amlexanox on patient PBMCs. Moreover, for the first time, the effects of amlexanox are explored *in vitro* on primary human B cells from patients with childhood-onset SLE (cSLE), SLE, pSjD and SSc.

## Patients and methods

### Patients and healthy controls

Female patients classified as cSLE or SLE according to the 1982 ACR revised criteria,^13^ as pSjD according to the criteria by *Vitali et al*^14^ and as SSc according to the ACR/EULAR 2013 criteria,^15^ as well as healthy controls (HC), were recruited at the Erasmus University Medical Center, Rotterdam, Netherlands. Demographics, clinical characteristics and type I IFN activation status of subjects included in the B cell differentiation assays are presented in online supplemental table 1. Low activity of the type I IFN system was confirmed in HC samples by analysis of whole blood MxA levels.^16^ The Rotterdam Medical Ethical Review Committee approved the study (MEC-2011–116; MEC-2016–202; MEC2019-0412), and all participants gave written informed consent.

### Blood sampling and processing

PBMCs were isolated from sodium-heparin tubes (Greiner Bio-One) through density gradient centrifugation using Ficoll-Paque PLUS (GE HealthCare) and cryopreserved in 50% RPMI-1640, 40% foetal bovine serum (FBS) (both Gibco) and 10% DMSO (Sigma-Aldrich) until further analysis. PAXgene RNA tubes (PreAnalytiX) were used for whole blood RNA analyses.

### Innate immune pathways stimulation

PBMCs were seeded in 96-wells round bottom plates at 5×10^6^ /mL in 100 µL RPMI-1640 medium with 10% or 0.5% FBS (both Gibco) and 0.05% penicillin/streptomycin (Lonza). Cells were then incubated with 75 µg/mL amlexanox (LKT labs) for 60 min and either harvested directly or stimulated further. The amlexanox concentration was decided by dose titrations (data not shown). For stimulations, Imiquimod (IQ) (1 µg/mL), CpG (1 µg/mL), PolyI:C (40 µg/mL), as well as G3-YsD (2.5 ng/mL) and 3p-hpRNA (2.5 ng/mL) through LyoVec (all Invivogen) transfection were added, following manufacturer’s protocol. In supernatant transfer experiments, freshly isolated PBMCs were seeded at 6 × 10^6^ /mL in 100 µL medium, incubated for 30 min with an anti-IFNAR antibody (5 µg/mL; MMHAR-2, Merck), and harvested supernatant was then added at a ratio of 1:4. Cells were incubated for 5 hours, and the expression of *MX1* and *BAFF* was measured by qPCR.

### B cell differentiation assay

CD19^+^B cells and memory B cells were isolated by magnetic bead separation using an autoMACS pro instrument (Miltenyi Biotec). B cell differentiation assays were performed as previously described.^17^ Briefly, irradiated NIH3T3 fibroblasts expressing human CD40L^18^ at density 1 × 10^6^ /mL in 100 µL medium were co-cultured in 96 wells flat-bottom plates for 6 days with total CD19^+^B cells or memory B cells at 0.5 × 10^5^ /mL in 100 µL in the presence of rhIL-21 (50 ng/mL; ThermoFisher) and the presence or absence of amlexanox (10 or 5 µg/mL; LKT labs), IFN-alpha 2b (200 IU/mL; PBL Assay Science) and BAFF (125 ng/mL; Peprotech). Cultured cells were analysed by flow cytometry as detailed below, and cell supernatants were stored at −80°C.

### Flow cytometry

In B cell culture experiments, cells were stained for 20 min in 10 µL Brilliant Stain Buffer Plus (BD Biosciences) and 5 µL True Stain Monocyte Blocker (BioLegend) with Apotracker Green for viability and antibodies targeting CD38 (APC/Fire 810, HIT2), CD27 (BV510, O323; all BioLegend), IgD (PE-Cy7, IA6-2), CD19 (APC, HIB19), CD20 (BUV563, 2H7), IgG (BV605, G18-145), CD45 (BV711, HI30), CD138 (BV421, MI15) and CD3 (BUV805, HIT3α; all BD Biosciences). To assess proliferation, isolated CD19^+^B cells were stained with 2.5 µM CellTrace Yellow (Thermo Fisher Scientific) according to manufacturer’s protocol. Cells were analysed on a 5-laser Cytek Aurora instrument (Cytek Biosciences). Data were analysed using OMIQ (www.omiq.ai; Dotmatics).

To analyse basal pTBK1 levels, PBMCs were stained with antibodies against BDCA-4 (PE, AD5-17F6, Miltenyi Biotec), CD123 (PE-Cy7, 6H6, eBioscience), CD3 (PerCP-Cy5, SK7), CD14 (APC-Cy7, MφP9) and CD19 (APC, SJ25C1; all BD Biosciences) and then fixed and permeabilised using a permeabilisation buffer set (eBioscience). Subsequently, cells were stained intracellularly with anti-pTBK1/NAK (Ser172) (D52C2; Cell Signaling Technology) followed by incubation with anti-rabbit antibody (AF488, Invitrogen) for detection of the pTBK1/NAK antibody and measured on a FACSCanto II (BD Biosciences). Data were analysed using FlowJo software (BD Life Sciences).

### Immunohistochemistry

Minor salivary gland (MSG) biopsies were obtained during diagnostic work-up of pSjD. Immunohistochemistry was performed with an automated, validated and accredited staining system (Ventana Benchmark ULTRA; Ventana Medical Systems) using ultraview or optiview universal DAB detection kit. In brief, following deparaffinisation and heat-induced antigen retrieval, the tissue samples were incubated according to their optimised time with the antibody of interest (online supplemental table 2). Incubation was followed by haematoxylin II counter stain for 12 min and then a blue colouring reagent for 8 min according to manufacturer’s instructions (Ventana Medical Systems).

### RT-PCR

RNA from PBMCs was isolated using RNeasy Mini Kits (Qiagen) followed by reverse transcription to cDNA using cDNA kits (Applied Biosystems). RNA from PAXgene RNA tubes was isolated according to the manufacturer’s protocol. RT-PCR analysis was performed on a Quantstudio 5 Real-Time PCR system using predesigned primer-probe sets (Applied Biosystems). Fold change values were determined from normalised CT values from household gene *ABL1* using the 2^-ΔCT^ method. Type I IFN scores were calculated as previously described,^19^ based on the expression of *IFI44*, *IFI44L*, *IFIT1*, *IFIT3*, *MX1* and *LY6E*.

### ELISA and cell reporter assay

IgG and IgM were measured in supernatants by human uncoated ELISA kits (Invitrogen) according to manufacturer’s instructions using 96-well Nunc MaxiSorp plates (Thermo Fisher Scientific). Supernatants were diluted 1:20 for measuring IgG and 1:50 for IgM. All samples were tested in duplicates. Whole blood MxA was measured using an enzyme immunoassay as previously described.^16^ Type I IFN activity was measured using HEK-Blue IFN-α/β cells (Invivogen) according to manufacturer’s protocol.

### Statistical analyses

For comparison of more than two groups, repeated measures one-way analysis of variance with Dunnett’s or Tukey’s multiple comparisons test was used. For comparison of two paired measurements, a two-tailed paired t-test was used. Spearman coefficient was employed for correlation analyses. Values of p<0.05 were considered significant and adjusted p values <0.05 in the case of multiple comparisons. Statistical analyses were carried out using Prism v 9.5.1 (GraphPad Software).

## Results

### Amlexanox inhibits the production and downstream effects of type Iinterferon induced through endosomal and cytoplasmic routes

**Figure 1:**
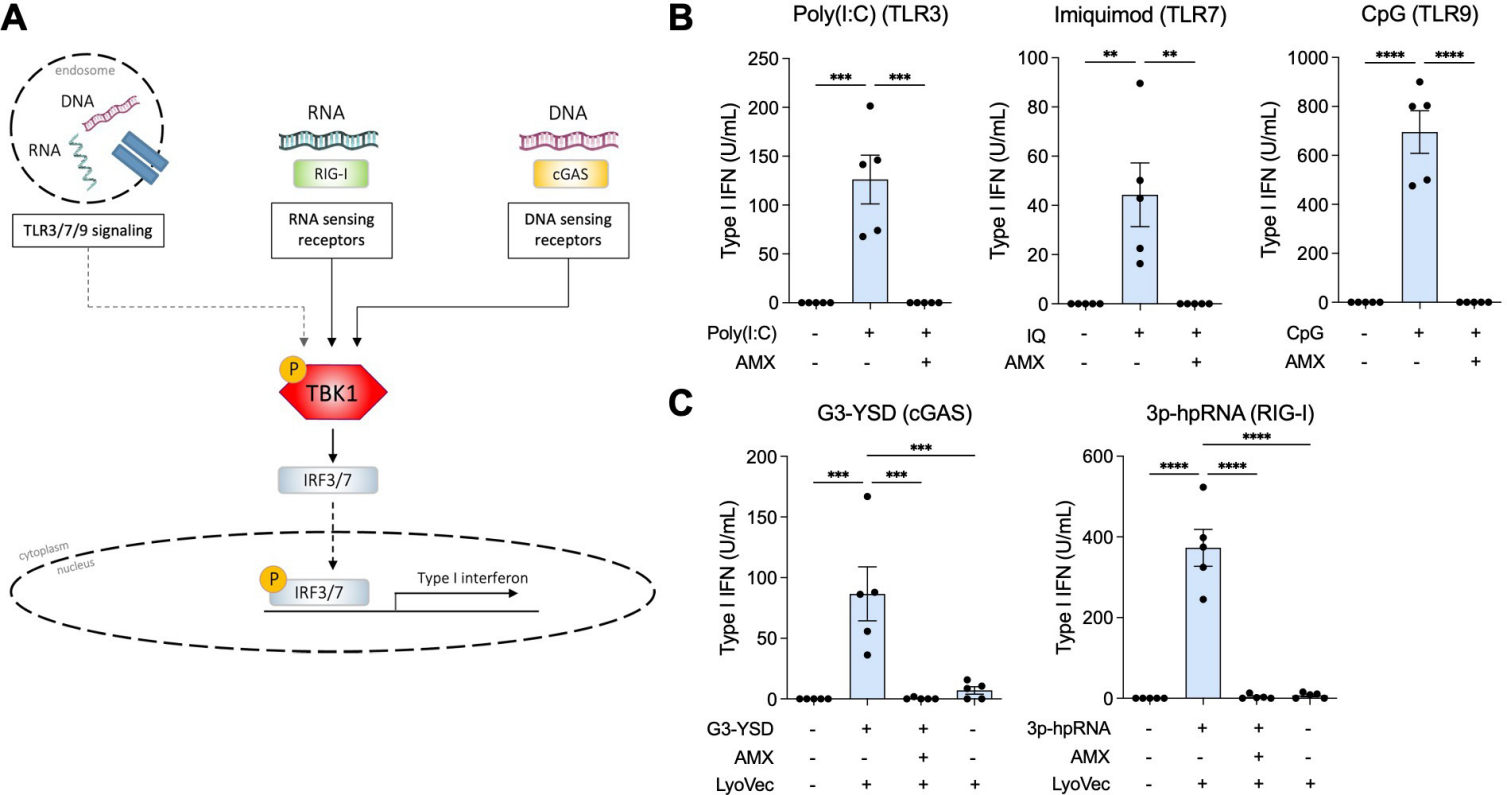
Amlexanox inhibits production of type I interferon (IFN) induced through endosomal and cytoplasmic routes. (**A**) Schematic overview of investigated endosomal and cytosolic pathways that signal through TANK-binding kinase 1 (TBK1). The dotted line represents the TLR7/9 pathway, in which the role of TBK1 is less well characterised. (**B**) Induction of type I IFN protein production by PBMCs determined by a HEK-Blue IFN-a/b reporter cell line after 16 hours incubation with or without amlexanox (AMX, 75 µg/mL) following stimulation of endosomal Toll-like receptors 3, 7 and 9, as well as (**C**) cytosolic receptors cGAS and RIG-I. Results are presented as mean±SEM **p<0.01, ***p<0.001 and ****p<0.0001 (repeated measures one-way analysis of variance with Tukey’s multiple comparisons test).

To assess the effect of amlexanox on type I IFN production, we investigated TBK1 signalling routes using a culture system with total PBMCs from HC. As such, we stimulated cells with agonists targeting endosomal TLRs, and cytosolic RLRs and DSRs were added with or without pre-incubation with amlexanox (figure 1A). Amlexanox inhibited the production of type I IFN induced by TLR3, TLR7 and TLR9 agonists (figure 1B), as well as type I IFN induced by agonists targeting cyclic GMP-AMP synthase (cGAS; agonist G3-YSD) and retinoic acid-inducible gene I (RIG-I; agonist 3p-hpRNA) (figure 1C). All agonists efficiently induced production of type I IFN, while spontaneous production of type I IFN or production induced by the transfection reagent LyoVec was not observed (figure 1B,C). Cell viability was not affected by amlexanox (online supplemental figure 1). To exclude possible effects of amlexanox downstream of the IFNAR, PBMC cultures were also stimulated with IFN-alpha 2b (online supplemental figure 2). Type I IFN-induced gene transcription of *MX1* and *BAFF* was not affected by the addition of amlexanox, thus indicating that amlexanox does not affect signalling downstream of the IFNAR.

**Figure 2:**
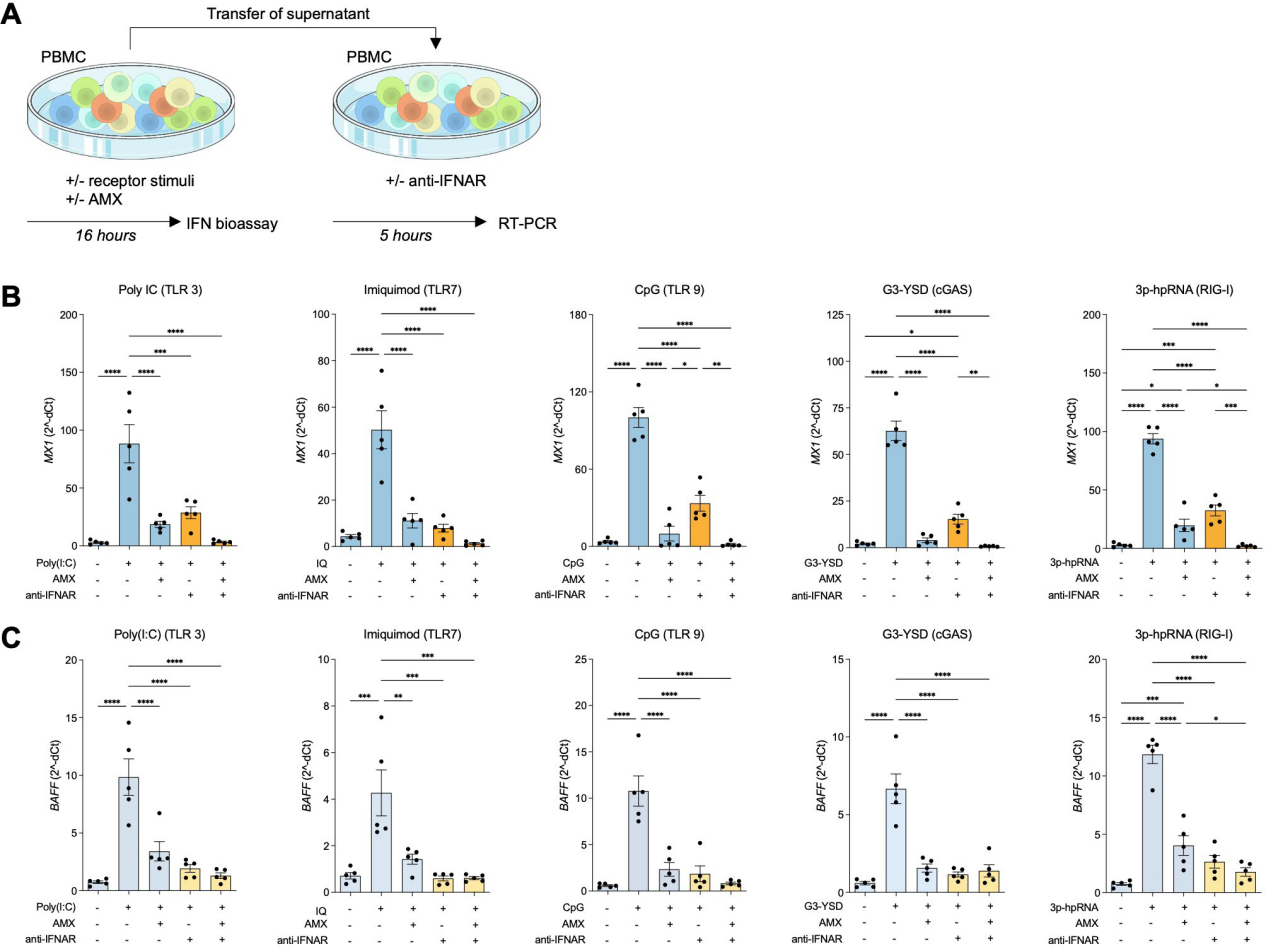
Supernatants from PBMC cultures pretreated with amlexanox do not induce expression of *MX1* and *BAFF*. (**A**) Supernatants from experiments presented in figure 1B,C were transferred to freshly isolated PBMCs, incubated for 5 hours and analysed by qPCR. (**B**) Expression of the type I interferon stimulated gene *MX1* and (**C**) *BAFF* induced by supernatants. Orange and yellow bars indicate the addition of an anti-interferon-α/β receptor (IFNAR) antibody. Amlexanox, AMX. Results are presented as mean±SEM *p<0.05 **p<0.01, ***p<0.001 and ****p<0.0001 (repeated measures one-way analysis of variance with Tukey’s multiple comparisons test).

To further substantiate that effective inhibition of type I IFN activity can be achieved by amlexanox, we transferred supernatants from the previous experiments to freshly isolated PBMCs and assessed induction of *MX1* and *BAFF* (figure 2A). A monoclonal antibody blocking the IFNAR was added as a control. Induced transcription of *MX1* and *BAFF* was efficiently reduced by supernatants of the amlexanox treated cultures, while supernatants from PBMCs to which the TLR, cGAS and RIG-I agonists had been added alone exhibited clear induction of the IFN stimulated genes (figure 2B,C). In most conditions, the effect of amlexanox was comparable to that of IFNAR blockade. However, in the RIG-I stimulated condition, inhibition of *MX1* and *BAFF* transcription by amlexanox alone was not complete, and a combined effect of amlexanox and IFNAR blockade was observed. In contrast, induction of *MX1* was more efficiently inhibited by amlexanox than by IFNAR blockade in the TLR9 stimulated condition.

### Amlexanox downregulates *MX1* transcription in PBMCs from patients with systemic autoimmune diseases with a high type I interferon score

**Figure 3:**
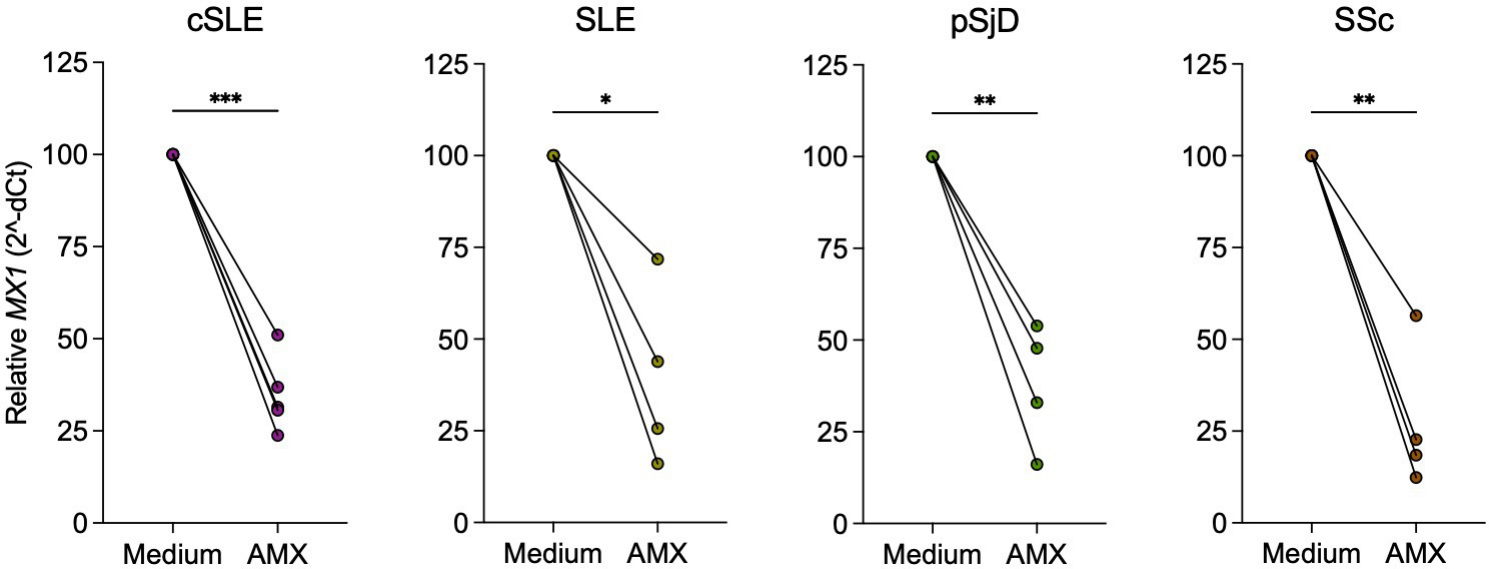
Amlexanox inhibits spontaneous interferon-stimulated gene transcription in PBMC from patients with systemic autoimmune diseases with a high type I interferon activation. PBMCs from patients with childhood-onset systemic lupus erythematosus (SLE) (cSLE, n=5), SLE (n=4), primary Sjögren’s disease (pSjD, n=4) and systemic sclerosis (SSc, n=4) and a high type I interferon score were incubated with amlexanox (AMX, 75 µg/mL) or culture medium for 5 hours and *MX1* expression measured by qPCR. Expression of *MX1* is shown relative to the control condition. *p<0.05 **p<0.01 and ***p<0.001 (paired t test).

PMBCs from patients with SLE, pSjD and SSc with a high type I IFN score exhibit spontaneous *in vitro* transcription of type I IFN induced genes such as *MX1*.^20^ To assess whether amlexanox could downregulate transcription of the prototypic type I IFN induced gene *MX1*, we selected patients with a high type I IFN activity and subjected their PBMCs to amlexanox or control medium for 5 hours. Addition of amlexanox to the patient cells resulted in significant reduction of *MX1* transcription (figure 3). A uniform pattern was observed in cells from patients with cSLE, SLE, pSjD and SSc, indicating that amlexanox may have a beneficial effect in lowering type I IFN activity in these diseases.

### TANK-binding kinase 1 is expressed in inflamed salivary gland tissue from patients with Sjögren’s disease

**Figure 4:**
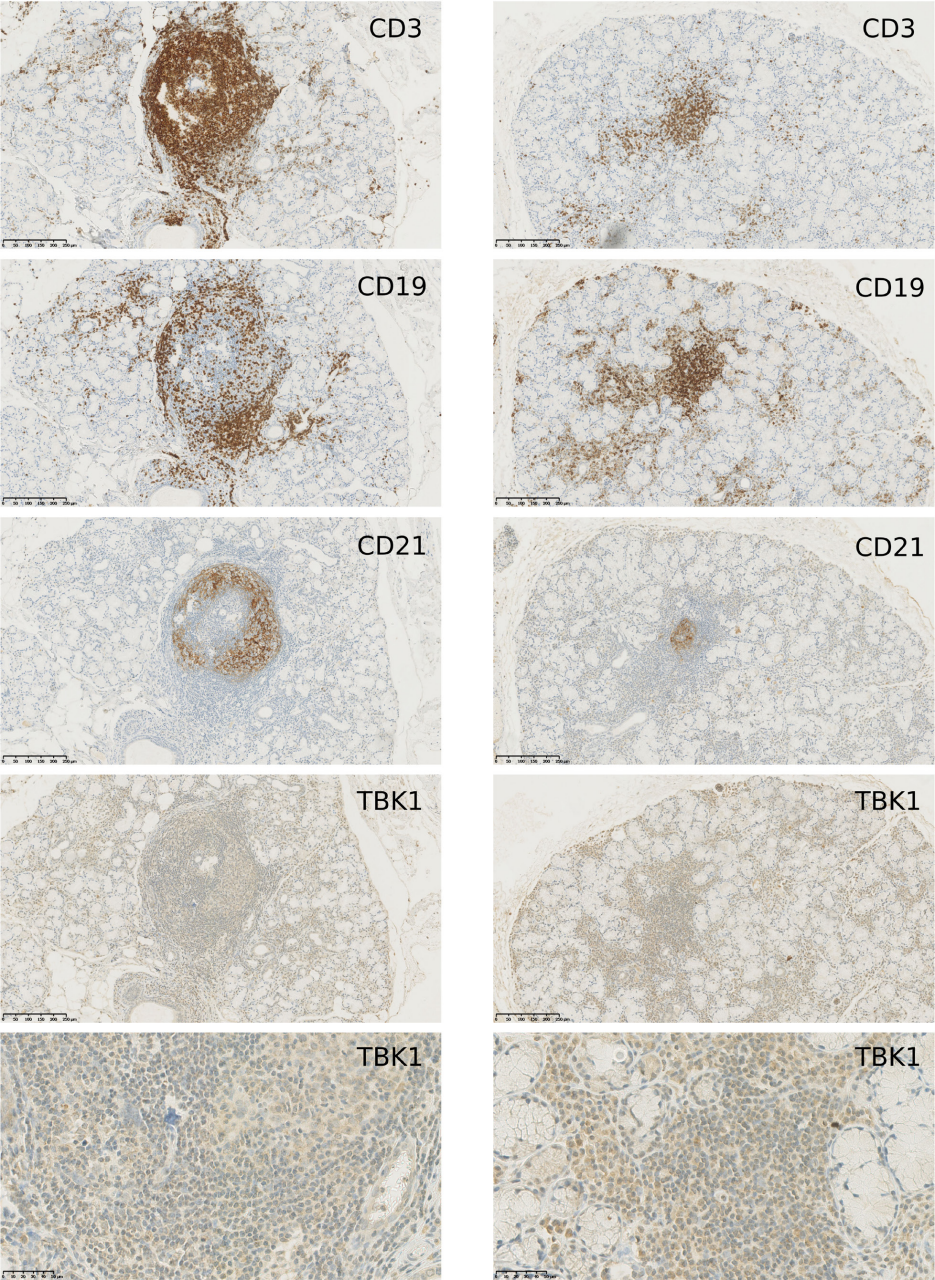
TANK-binding kinase 1 (TBK1) is expressed inside ectopic germinal centre formations and the parenchyma in minor salivary gland biopsies from patients with primary Sjögren’s disease. Minor salivary gland biopsies (n=20) were stained for T cells (CD3), B cells (CD19), follicular dendritic cells in germinal centre-like structures (CD21) and TBK1. Representative pictures are shown at ×10 magnification and ×40 magnification for TBK1 in a focal infiltrate on the last row.

Production of type I IFN partly occurs in the inflamed target tissue in systemic autoimmune diseases.^21^ The study of patient tissue requires biopsy material, which was accessible to us in the form of MSG biopsies from patients with pSjD. Indeed, local production of type I IFN has previously been described in MSG biopsies.^22^ To further substantiate the relevance of TBK1 inhibition in systemic diseases with a high type I IFN activity, we explored the expression of the TBK1 protein in inflammatory infiltrates of MSG biopsies from patients with pSjD. Tissue sections from patients with pSjD containing focal lymphocytic infiltrates were stained to identify CD3^+^T cells, CD19^+^B cells and TBK1 expression, while CD21, which is present on follicular dendritic cells, was used as a proxy marker for ectopic GCs^23^ (figure 4, online supplemental table 2). GCs are essential for activation, proliferation and differentiation of mature B cells, and the presence of GCs in MSG biopsies has been associated with higher disease activity in pSjD.^24 25^ In GCs, TBK1 expression was observed concomitant with CD3 and CD19. In foci and the parenchyma, expression of TBK1 was detected, which would be expected also in healthy tissue since TBK1 is ubiquitously expressed in haematopoietic and non-haematopoietic cells. A weak but significant correlation was observed between a quantified staining score for TBK1 and the focus score in n=20 MSG biopsies (online supplemental figure 3).

### Amlexanox inhibits B cell proliferation and differentiation, as well as immunoglobulin production

**Figure 5:**
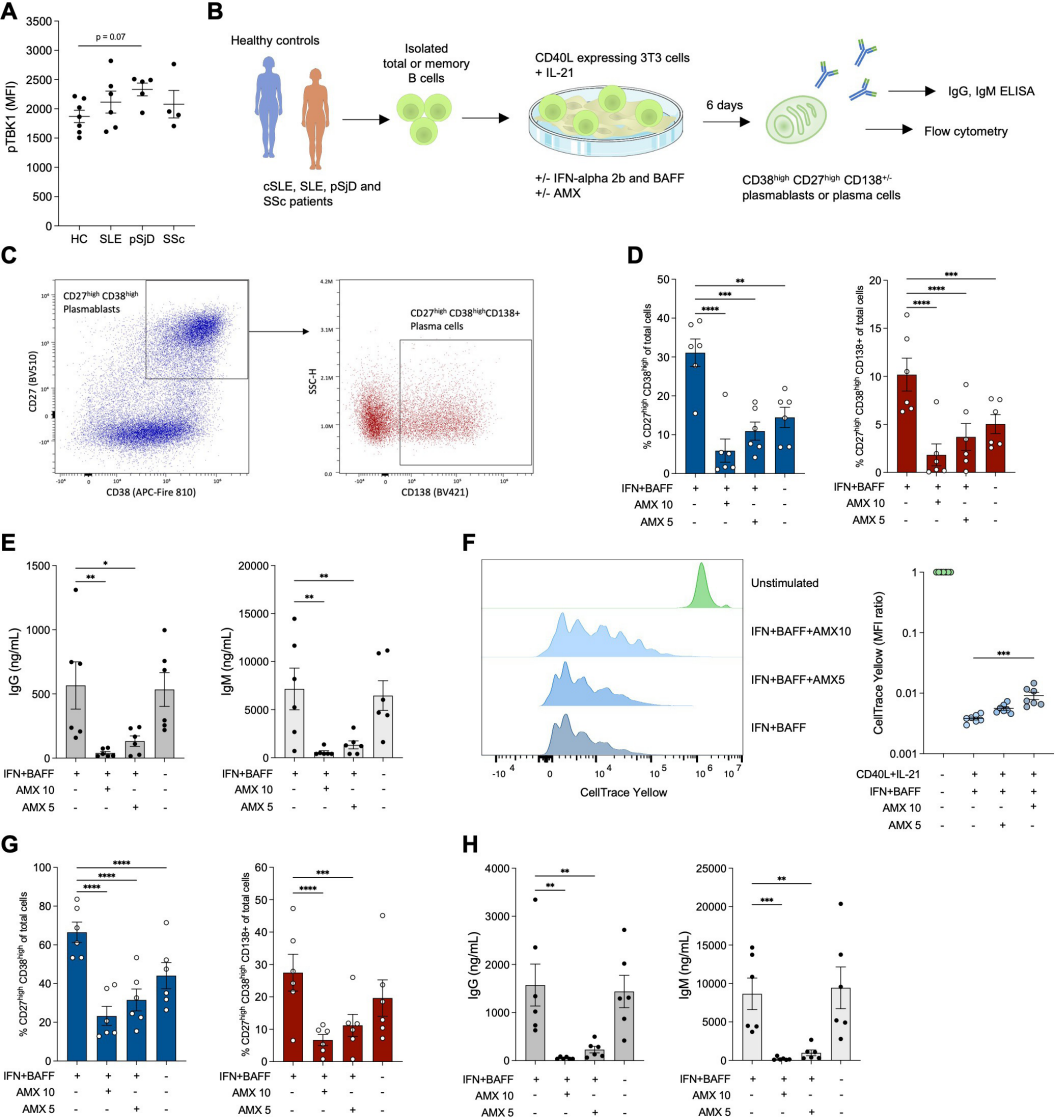
Amlexanox inhibits proliferation and differentiation of B cells into plasmablasts as well as immunoglobulin production *in vitro*. (**A**) Phosphorylated TANK-binding kinase 1 (pTBK1) was measured in CD19^+^B cells from healthy controls (HC) and patients with systemic lupus erythematosus (SLE), primary Sjögren’s disease (pSjD) and systemic sclerosis (SSc) by phosphoflow. Median fluorescent intensity (MFI) with subtracted isotype control is shown. (**B**) Experimental setup of the B cell differentiation experiments. (**C**) Gating strategy for CD27^high^CD38^high^ plasmablasts and CD27^high^CD38^high^CD138^+^ plasma cells. (**D**) Percentage of CD27^high^CD38^high^ plasmablasts and CD27^high^CD38^high^CD138^+^ plasma cells out of total cells at day 6 in conditions with or without added interferon-alpha 2b (IFN), B cell activating factor (BAFF) and amlexanox (AMX) at two different concentrations (10 µg/mL and 5 µg/mL). (**E**) Concentrations of IgG and IgM in the corresponding supernatants. (**F**) Proliferation of B cells assessed by CellTrace Yellow staining to trace cell divisions. The graph to the left shows representative fluorescence at day 6, with unstimulated B cells as reference. The graph to the right shows quantified MFI relative to the corresponding unstimulated condition. (**G**) Percentage of CD27^high^CD38^high^ plasmablasts and CD27^high^CD38^high^CD138^+^ plasma cells out of total cells at day 6 and (**H**) levels of IgG and IgM in cultures started with memory B cells. Results are presented as mean±SEM *p<0.05 **p<0.01, ***p<0.001, and ****p<0.0001 (A) Kruskal-Wallis with Dunn’s multiple comparisons test; (D,E,F,G,H) repeated measures one-way analysis of variance ANOVA with Dunnett’s multiple comparisons test).

Considering the key role of B cells in the immunopathology of systemic autoimmune diseases and the fact that TBK1 expression can be detected in B cells in inflamed tissue, we next explored potential B cell specific effects of TBK1 inhibition. We first measured levels of phosphorylated TBK1 (pTBK1) in total B cells from patients with SLE, pSjD and SSc by phosphoflow. A tendency of higher pTBK1 levels was noted in patients compared with HC, although not significant (figure 5A). We next hypothesised that TBK1 inhibition may interfere with differentiation of B cells towards antibody-secreting cells. To investigate B cell differentiation under T follicular helper-like conditions, we used MACS-purified CD19^+^total B cells from healthy controls with low blood MxA levels and cultured these together with CD40L-expressing NIH3T3^18^ feeder cells and IL-21 as previously described.^17^ To mimic an autoimmune setting, IFN-alpha 2b and BAFF were added to the cultures (figure 5B). At day 6, we assessed differentiation into CD27^high^CD38^high^ plasmablasts and CD27^high^CD38^high^CD138^+^ plasma cells and measured immunoglobulin (Ig) levels in culture supernatants (figure 5C, complete gating strategy in online supplemental figure 4). Addition of amlexanox at 10 or 5 µg/mL significantly decreased differentiation into plasmablasts and plasma cells (figure 5D). Correspondingly, production of IgG and IgM was lower in the amlexanox-treated conditions (figure 5E). Addition of IFN-alpha 2b and BAFF to the cultures enhanced differentiation of B cells into plasmablasts (figure 5D) compared with cultures without addition of IFN-alpha 2b, BAFF or amlexanox, whereas production of IgG and IgM was unaffected. In line with these data, a gradual inhibition of B cell differentiation was confirmed by CellTrace staining which traces cell divisions, reaching significance at the 10 µg/mL amlexanox concentration (figure 5F).

To better understand at which stage of differentiation amlexanox elicits its effect, we next started the same cultures but instead using memory B cells. Differentiation into plasmablasts and plasma cells, as well as IgG and IgM production, was inhibited by amlexanox in a similar manner to that observed for CD19^+^total B cells (figure 5G,H). As could be anticipated, relative frequencies of differentiated B cells as well as Ig production were higher in these cultures compared with those started using CD19^+^total B cells.

### Plasmablast differentiation and immunoglobulin production are inhibited by amlexanox in systemic autoimmune diseases with a high type I interferon activity

**Figure 6:**
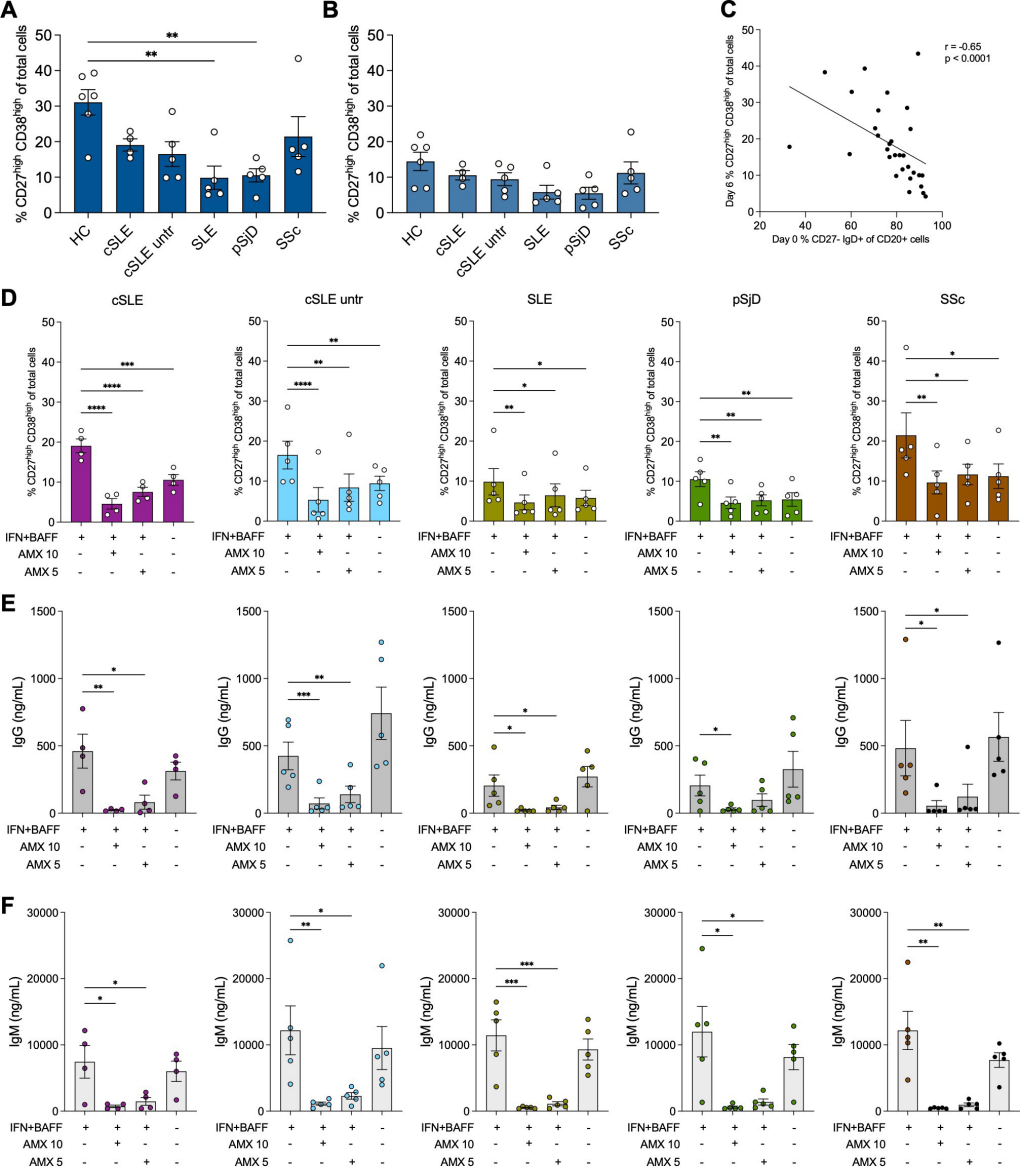
Plasmablast differentiation and immunoglobulin production are inhibited by amlexanox in systemic autoimmune diseases with a high type I interferon activity. (**A**) Percentage of CD27^high^CD38^high^ plasmablasts at day 6 in cultures with added interferon-alpha 2b (IFN) and B cell activating factor (BAFF), and (**B**) without added IFN and BAFF, for healthy controls (HC), childhood-onset systemic lupus erythematosus (cSLE), SLE, primary Sjögren’s disease (pSjD) and systemic sclerosis (SSc). (**C**) Relative frequencies of naïve B cells at the start of the cultures and plasmablast frequencies at day 6 for all samples in the condition with added IFN and BAFF (Spearman correlation). (**D**) Percentage of CD27^high^CD38^high^ plasmablasts and out of total cells at day 6 in conditions with or without added IFN, BAFF and amlexanox (AMX) at two different concentrations (10 µg/mL and 5 µg/mL). (**E**) Concentrations of IgG and (**F**) IgM in the corresponding supernatants. Results are presented as mean±SEM *p<0.05 **p<0.01, ***p<0.001 and ****p<0.0001 (repeated measures one-way analysis of variance with Dunnett’s multiple comparisons test).

To corroborate our findings in experimental conditions more relevant to systemic autoimmune diseases, we next performed the same B cell culture experiments using B cells isolated from patients with cSLE, SLE, pSjD and SSc. The patients were selected to have high type I IFN activity measured by an IFN score as well as blood MxA levels^16^ and mild immunomodulatory treatment (online supplemental table 1). In these experiments, we had the possibility to include samples from cSLE patients taken at diagnosis before the start of any immunomodulatory treatment (indicated as cSLE untr). Like the results from HC cells, the addition of IFN-alpha 2b and BAFF to patient cells increased differentiation into plasmablasts (figure 6A,B). To our surprise, cells from SLE and pSjD patients differentiated less into plasmablasts compared with cells from HC (figure 6A). However, this could be explained by disease-specific differences in B cell subpopulation frequencies, since an inverse correlation was observed between relative frequencies of naïve B cells at the start of the cultures and plasmablast frequencies at day 6 (figure 6C, online supplemental figures 5 and 6), that is, patient samples contained higher relative frequencies of B cells at early stages of differentiation and therefore did not differentiate into plasmablasts as quickly as samples from HC. In all diseases, amlexanox at the 10 or 5 µg/mL concentration significantly inhibited plasmablast differentiation and IgG and IgM production, except for the lower amlexanox concentration and IgG production in pSjD (figure 6D–F). Corresponding data for differentiation into plasma cells are shown in online supplemental figure 7. Starting cSLE cultures with memory B cells showed a similar pattern to that observed in HC (online supplemental figure 8). In summary, these data support an inhibitory effect of amlexanox on production of type I IFN as well as on B cell differentiation and antibody production in cells from patients with systemic autoimmune diseases, which may be advantageous in terms of therapeutic strategy.

## Discussion

Inhibition of TBK1 has been repeatedly suggested as a theoretical option for the treatment of systemic autoimmune diseases, primarily owing to the key role of TBK1 upstream of type I IFN expression.^2026^,^28^ Here, we describe for the first time indications of beneficial effects on immunopathological processes by the small molecule inhibitor of TBK1 amlexanox in samples from patients with SLE, pSjD and SSc.

Several lines of evidence suggest the importance of TBK1 in systemic autoimmune diseases. Gene expression of *TBK1* has been reported at higher levels in leukocytes from SLE patients compared with controls, whereas expression of *IKBKE* coding for IKKε did not differ.^29^ Furthermore, expression of *TBK1* as well as its downstream mediators *IRF3* and *IRF7* was observed previously at higher levels in PBMCs from SLE, pSjD and SSc patients compared with controls, suggesting hyperactivity of the TBK1 signalling pathway in these diseases.^20^ In our study, amlexanox downregulated type I IFN production induced by endosomal TLRs and cytosolic RLRs and DSRs in PBMCs, as well as secondary induction of the IFN-stimulated genes *MX1* and *BAFF*. These data are in line with observations made in a lung epithelial carcinoma reporter cell line, in which amlexanox inhibited luciferase activity (used as surrogate marker for type I IFN production) induced by PolyI:C, 3p-hpRNA and 2′,3′-cGAMP.^28^ Correspondingly, another TBK1/IKKε inhibitor BX795 was shown by us to downregulate type I IFN production induced by Imiquimod, as well as spontaneous transcription of type I IFN stimulated genes *IFI44*, *IFI44L*, *IFIT1*, *IFIT3* and *MX1* in PBMCs from patients with SLE, pSjD and SSc.^20^ Moreover, BX795 lowered type I IFN production in PBMCs from patients with gain-of-function mutations in the stimulator of interferon genes (STING) protein, further suggesting the usefulness of TBK1/IKKε inhibition for treatment of interferonopathies.^30^ TBK1/IKKε are not classically described to be involved in the TLR7 and TLR9 signalling pathways, and therefore our data may seem surprising. However, it has been described that ligands which signal through MyD88 activate TBK1 and IKKε at least as robustly as PolyI:C^31^ and that TBK1/IKKε kinase activity seems to be required for TLR7-stimulated TANK SUMOylation and the consequent release of active TRAF6.^32^ TRAF6 is involved in TLR7/9-induced IRF7 activation. As such, inhibition of TBK1/IKKε may modulate TLR7/9-stimulated production of type I IFN, which could explain our observations.

Amlexanox has been studied in humans for its effects on metabolic disease. In a study of 42 obese patients with type 2 diabetes, amlexanox treatment administered as 50 mg tablets three times per day for 12 weeks resulted in a reduction of haemoglobin A1c levels and in a subgroup improved insulin sensitivity and hepatic steatosis.^12^ Importantly, this study indicated that amlexanox is well-tolerated, with a self-limiting rash not leading to drug discontinuation reported in one patient. Further support for favourable metabolic effects has been found using three different obese mouse models, in which amlexanox treatment resulted in reduced weight, insulin resistance, inflammation and steatosis.^11^ Moreover, amlexanox improved blood lipids in Western diet-fed *Ldlr-/-* mice, protecting against atherogenesis.^10^

Only one study has previously assessed amlexanox in the context of autoimmunity. In a collagen II-induced rheumatoid arthritis mouse model, amlexanox was reported to alleviate inflammatory response and joint activity.^33^ We here describe for the first time that PBMCs from patients with cSLE, SLE, pSjD and SSc and high type I IFN activity respond to amlexanox with downregulation of the IFN-stimulated gene *MX1*. This is desirable since blocking of type I IFN signalling is useful for the treatment of active SLE.^5^

Thus far, studies of the effect of TBK1 inhibition have not been performed in humans with systemic autoimmune diseases. However, TBK1/IKKε inhibition by Compound II was shown to ameliorate autoimmune disease phenotypes in the *Trex1^-/-^* mouse model for Aicardi–Goutières syndrome, decreasing IFN gene signature and autoantibody titres as well as increasing survival.^29^ Potential risks of TBK1 inhibition have been raised, such as increased susceptibility to infections, as well as a link between heterozygous loss-of-function mutations in TBK1 with neurodegenerative diseases.^34^ However, oral administration of amlexanox for 24 weeks indicated that the drug was not associated with higher numbers of infections.^12^

A role for TBK1 in adaptive immunity is emerging.^26^ Studies in a murine *Tbk1^-/-^* model on a *Tnf^-/-^* background (TBK1-deficient mice otherwise die in utero) showed impaired IgG responses to a DNA vaccine when TBK1-mediated signalling was not present in haematopoietic cells.^35^ In another study using mice with TBK1-deficient B cells, TBK1 was found to regulate signalling downstream of the B cell receptor and CD40.^6^ The GC-like B cell culture system used in our study relied on feeder cells expressing CD40L; wherefore it is tempting to speculate that effects seen by amlexanox may in part be explained by dysregulated signalling downstream of CD40. In murine models, TBK1 has been implicated downstream of CD40 by negatively regulating the signalling via the non-canonical NF-κB pathway in B cells.^6 8^ There are conflicting data regarding phosphorylation of TBK1 on anti-CD40 stimulation of B cells, with reports suggesting both increased^8^ and decreased^6^ pTBK1 levels on stimulation. Thus, how TBK1 is involved in CD40 signalling requires further research. We observed that amlexanox inhibited differentiation and proliferation into plasmablast-like cells as well as IgG and IgM production. This pattern was confirmed in B cells from patients with cSLE, SLE, pSjD and SSc. To our knowledge, inhibition of TBK1 in primary human B cells has not been previously studied. However, in diffuse large cell B cell lymphoma cell lines, small molecule inhibitors of TBK1/IKKε were shown to suppress survival.^36^ Interestingly, in line with this effect on survival, immunophenotypic characterisation of three TBK1-deficient humans revealed much lower plasma cell frequencies in the circulation compared with healthy individuals.^37^ In our study, B cell cultures were started both with total CD19^+^B cells and with memory B cells, revealing effects on B cell differentiation in both cell types by amlexanox. These results indicate the relevance of TBK1 activity also at later stages of B cell differentiation. Still, more mechanistic research is required to conclusively demonstrate the B cell intrinsic role of TBK1 in systemic autoimmunity.

Limitations of our study relate to the fact that amlexanox does not specifically inhibit only TBK1 but also the related kinase IKKε. However, in TBK1-deficient humans, a compensatory upregulation of IKKε has been described to provide a critical back-up mechanism which protects against diminished IFN responses.^38^ This suggests that simultaneous inhibition of both TBK1 and IKKε may in fact be desirable for treatment of interferonopathies. Other limitations of our study are the artificial nature of the *in vitro* B cell culture system, and the effects of amlexanox on other immune cell subsets or in murine models of autoimmune disease were not addressed. The B cell culture system did not encompass all autoimmune-related cytokines and did not allow for detailed mechanistic studies of TBK1 functions. Strengths of the study relate to the fact that results were corroborated using samples from patients with several systemic autoimmune diseases.

In conclusion, our data support dual inhibitory effects of amlexanox on type I IFN production and B cell differentiation *in vitro* using primary human cells. These novel observations suggest that inhibition of TBK1/IKKε by amlexanox may result in beneficial effects on key processes involved in systemic autoimmune diseases. However, *in vivo* effects of amlexanox were not assessed, wherefore its therapeutic potential is circumstantial and subject to more research. Drug repurposing of amlexanox could offer accelerated development and reduced risk compared with de novo synthesised TBK1/IKKε inhibitors.

## Supplementary material

10.1136/rmdopen-2024-005351 online supplemental figure 1

10.1136/rmdopen-2024-005351 online supplemental table 1

10.1136/rmdopen-2024-005351 online supplemental table 2

## Data Availability

All data relevant to the study are included in the article or uploaded as supplementary information.
