# Supplementary material for: Amlexanox inhibits production of type I interferon and suppresses B cell differentiation in vitro: a possible therapeutic option for systemic lupus erythematosus and other systemic inflammatory diseases
Source: RMD Open. 2025 May 7;11(2):e005351. doi: 10.1136/rmdopen-2024-005351 (PMC12060889; doi:10.1136/rmdopen-2024-005351)
Supplement: online supplemental figure 1 [file rmdopen-11-2-s001.pdf]

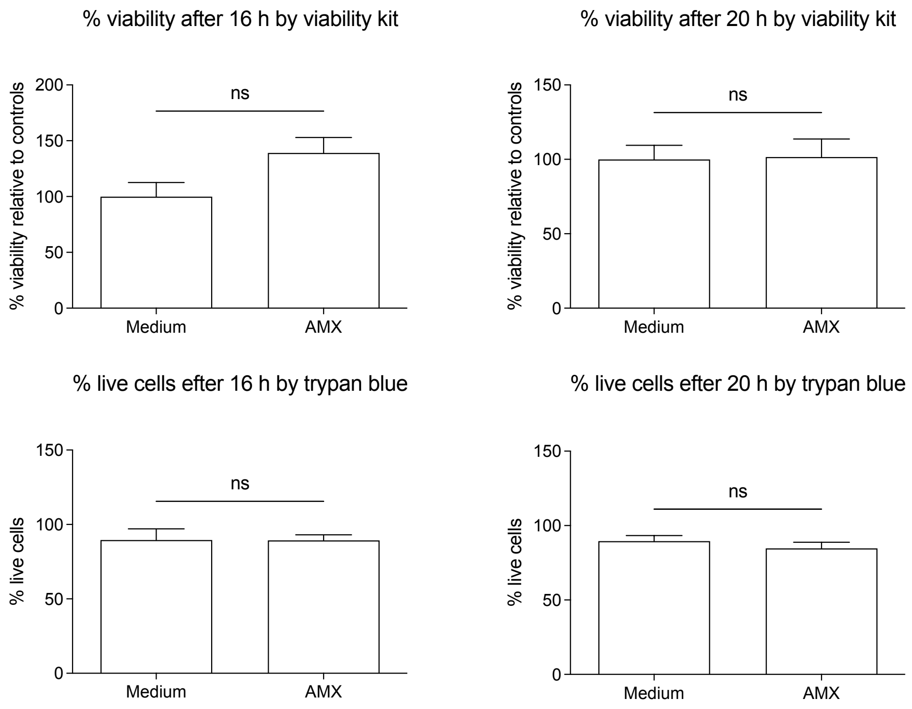

Supplementary figure 1. Amlexanox (AMX) 75 µg/mL does not affect viability of PBMCs after 16 and 20 hours. Cell viability was assessed both by a kit (Abcam; ab228554) shown on the upper row and by trypan blue staining shown on the lower row. Ns, not significant.

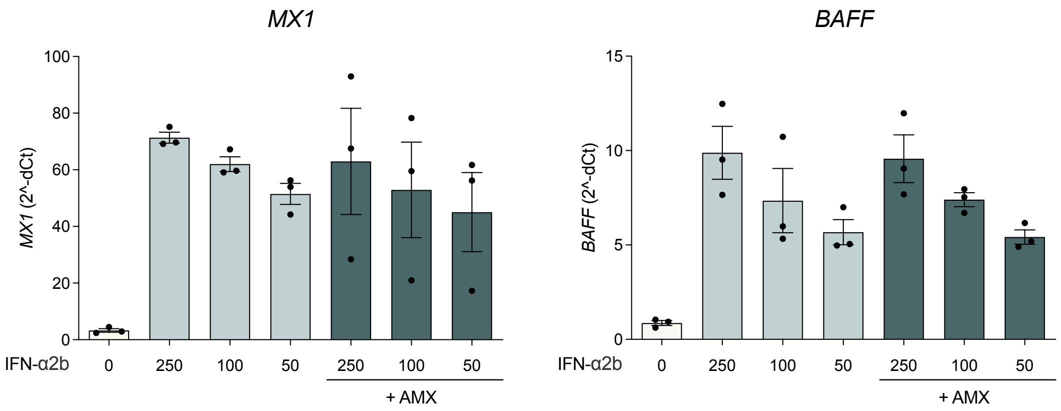

Supplementary figure 2. Amlexanox (AMX) has no effect downstream of the interferon alpha receptor. Relative *MX1* and *BAFF* gene expression in cell cultures treated with amlexanox (AMX) and stimulated with indicated IFN-alpha 2b concentrations (IU/mL).

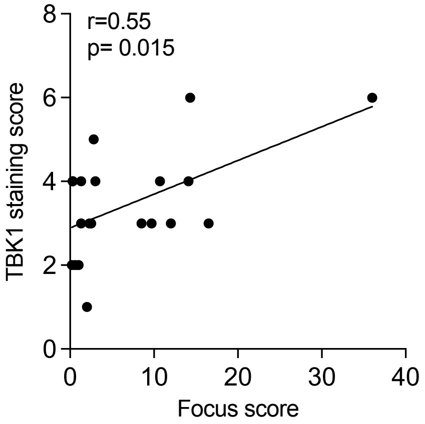

Supplementary figure 3. Intensity of Tank binding kinase 1 (TBK1) staining plotted versus focus score. Two investigators independently assessed intensity of the TBK1 staining on a scale from 1-3 in mononuclear cells in focal infiltrates, ducts and epithelium of n = 20 minor salivary gland biopsies from patients with primary Sjögren's disease. The sum of the scores (max 9) represents the mean. (Spearman correlation).

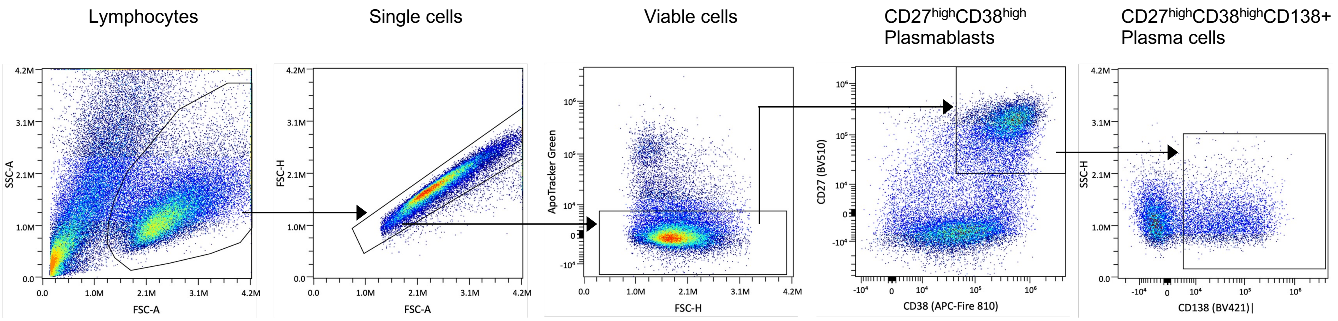

Supplementary figure 4. Gating strategy for plasmablasts and plasma cells in B cell *in vitro* cultures.

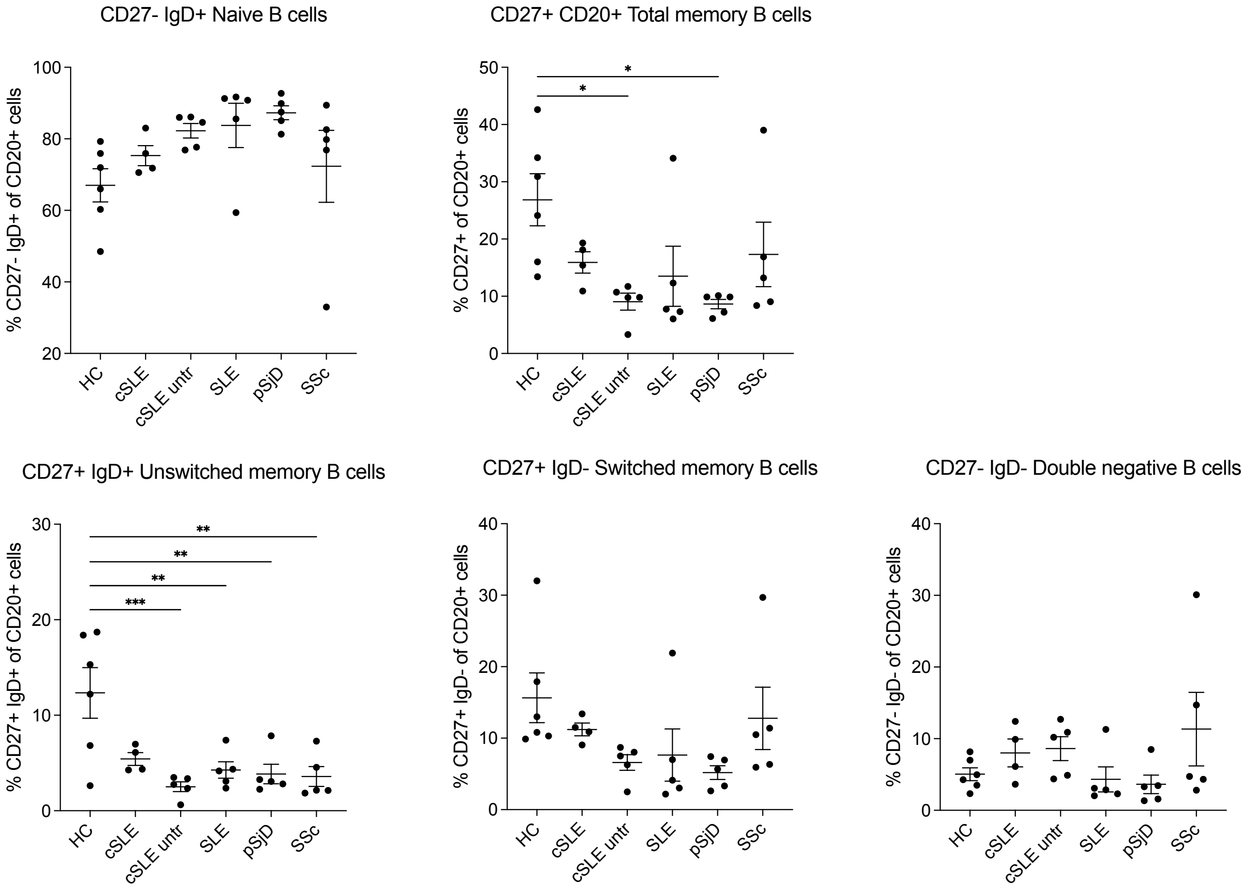

Supplementary figure 5. B cell subpopulation frequencies at day 0.

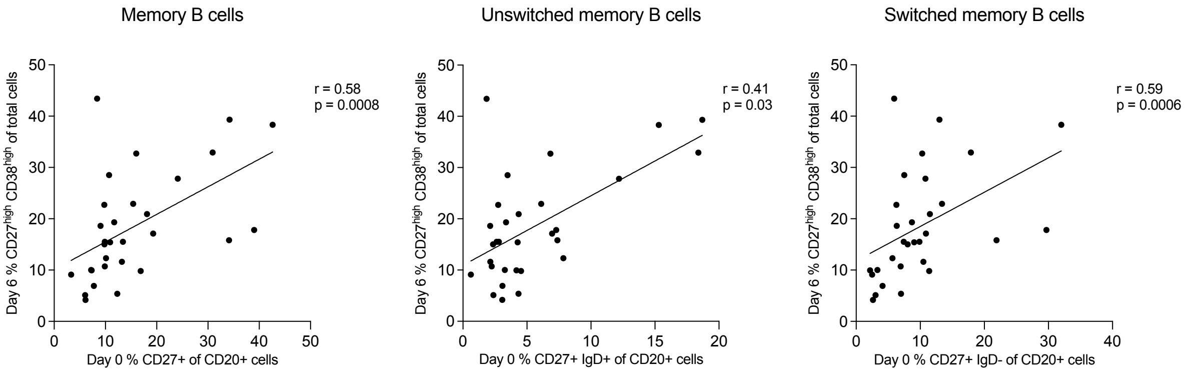

Supplementary figure 6. Spearman correlation between B cell subpopulation frequencies at day 0 and $CD27^{high}$ $CD38^{high}$ cell frequencies at day 6.

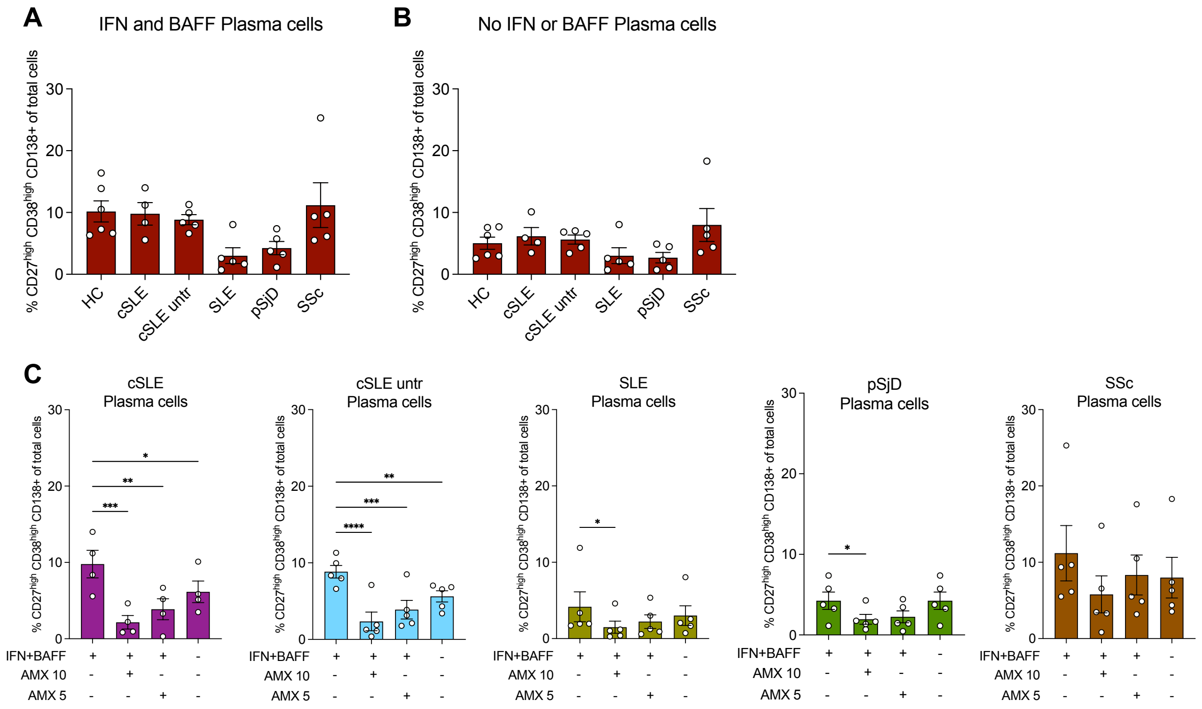

Supplementary figure 7. Plasma cell frequencies at day 6.

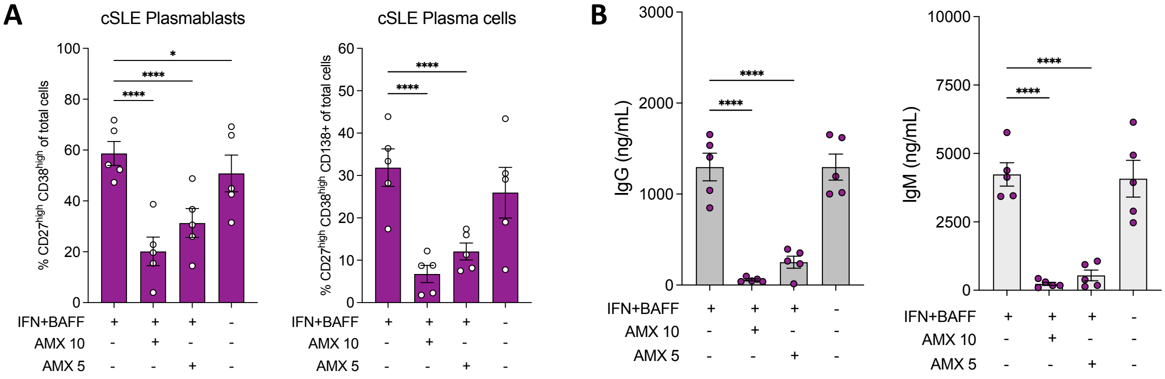

Supplementary figure 8. B cell differentiation and immunoglobulin production in memory B cell cultures of childhood-onset SLE patients.
