# Supplementary material for: Amlexanox inhibits production of type I interferon and suppresses B cell differentiation in vitro: a possible therapeutic option for systemic lupus erythematosus and other systemic inflammatory diseases
Source: RMD Open. 2025 May 7;11(2):e005351. doi: 10.1136/rmdopen-2024-005351 (PMC12060889; doi:10.1136/rmdopen-2024-005351)
Supplement: online supplemental table 1 [file rmdopen-11-2-s002.docx]

**Supplementary table 1.** Clinical characteristics and type I IFN activation status of subjects included in the B cell differentiation assays

|  | HC (n=6) | cSLE (n=4) | cSLE untr (n=5) | SLE (n=5) | pSjD (n=5) | SSc (n=5) |
| --- | --- | --- | --- | --- | --- | --- |
| **Demographics** |  |  |  |  |  |  |
| Female, n (%) | 6 (100) | 4 (100) | 5 (100) | 5 (100) | 5 (100) | 5 (100) |
| Age, years (mean ± SD) | 46.5 ± 19.5 | 15.3 ± 2.4 | 12.4 ± 2.1 | 38.2 ± 16.8 | 55.2 ± 8.2 | 58.4 ± 13.7 |
|  |  |  |  |  |  |  |
| **Patient characteristics** |  |  |  |  |  |  |
| Disease duration, years (mean ± SD) | n.a. | 2.5 ± 2.4 | 0 ± 0 | 13.4 ± 15.6 | 6.4 ± 4.4 | 16 ± 13.5 |
| Disease activity^a^ | n.a. | 4.0 ± 2.8 | 10.4 ± 5.7 | 2.0 ± 1.4 | 6.2 ± 4.0 | n.a. |
|  |  |  |  |  |  |  |
| **Medication status** |  |  |  |  |  |  |
| Corticosteroids, n (%) | 0 (0) | 0 (0) | 0 (0) | 0 (0) | 0 (0) | 0 (0) |
| csDMARDs, n (%) | 0 (0) | 4 (100) | 0 (0) | 4 (80) | 0 (0) | 1 (20) |
|  |  |  |  |  |  |  |
| **IFN activation status** |  |  |  |  |  |  |
| IFN score, normalized to control population | m.d. | 16.8 ± 2.3 | 17.8 ± 3.9 | 16.6 ± 0.7 | 19.5 ± 2.8 | 18.0 ± 8.1 |
| Blood MxA (μg/L) | 8 ± 14^b^ | 608 ± 209 | 490 ± 352 | 191 ± 106 | 286 ± 136 | 234 ± 127 |

^a^Disease activity determined by ESSDAI for pSjD and SLEDAI for SLE and cSLE

^b^Data is missing for one individual

n.a., not applicable; m.d., missing data; IFN, interferon; csDMARDs, conventional synthetic disease-modifying antirheumatic drugs; untr, untreated; HC, healthy controls; cSLE, childhood-onset systemic lupus erythematosus; SLE, systemic lupus erythematosus; pSjD, primary Sjögren’s disease; SSc, systemic sclerosis.
