# Supplementary material for: Amlexanox inhibits production of type I interferon and suppresses B cell differentiation in vitro: a possible therapeutic option for systemic lupus erythematosus and other systemic inflammatory diseases
Source: RMD Open. 2025 May 7;11(2):e005351. doi: 10.1136/rmdopen-2024-005351 (PMC12060889; doi:10.1136/rmdopen-2024-005351)
Supplement: online supplemental table 2 [file rmdopen-11-2-s003.docx]

**Supplementary table 2.** Antibodies used for immunohistochemistry

| **Antibody** | **Type** | **Concentration** | **Company** | **Clone** | **Procedure** | **Incubation time** |
| --- | --- | --- | --- | --- | --- | --- |
| TBK1 | Anti-Mouse | 1.0 mg/ml | ThermoFisher | *polyclonal* | Ultraview CC1 64' | 120 minutes |
| CD21 | Anti-Mouse | 133 µg/ml | Cell Marque | 2G9 | Ultraview CC1 32' | 68 minutes |
| CD3 | Anti-Rabbit | 1.4 µg/ml | Ventana | 2VG6 | Optiview CC1 32' | 32 minutes |
| CD19 | Anti-Mouse | 1/1500 | Invitrogen | LE-CD19 | Optiview CC1 32' | 48 minutes |
